# Cross Talk between Proliferative, Angiogenic, and Cellular Mechanisms Orchestred by HIF-1*α* in Psoriasis

**DOI:** 10.1155/2015/607363

**Published:** 2015-06-07

**Authors:** Azael Torales-Cardeña, Isaí Martínez-Torres, Sandra Rodríguez-Martínez, Fernando Gómez-Chávez, Juan C. Cancino-Díaz, Ernesto A. Vázquez-Sánchez, Mario E. Cancino-Díaz

**Affiliations:** ^1^Immunology Department, National School of Biological Sciences, National Polytechnic Institute, Plan de Ayala y Prolongación de Carpio S/N, Colonia Santo Tomás, Miguel Hidalgo, 11340 Mexico City, DF, Mexico; ^2^Microbiology Department, National School of Biological Sciences, National Polytechnic Institute, Plan de Ayala y Prolongación de Carpio S/N, Colonia Santo Tomás, Miguel Hidalgo, 11340 Mexico City, DF, Mexico

## Abstract

Psoriasis is a chronic inflammatory skin disease where the altered regulation in angiogenesis, inflammation, and proliferation of keratinocytes are the possible causes of the disease, and the transcription factor “hypoxia-inducible factor 1-alpha” (HIF-1*α*) is involved in the homeostasis of these three biological phenomena. In this review, the role of HIF-1*α* in the cross talk between the cytokines and cells of the immunological system involved in the pathogenesis of psoriasis is discussed.

## 1. The Psoriasis

Psoriasis is a chronic inflammatory disease of the skin with unknown etiology but associated with angiogenesis and with proliferative and immunological dysfunction, all manifested in the skin. Global epidemiology has suggested that the incidence of psoriasis varies according to age and geographic region; it is estimated that 2-3% of the world population have psoriasis being more frequent in the Caucasian population with an incidence of about 100,000 people per year [1, 2]. There are several types of psoriasis, but all are characterized by skin thickening, erythema, and pustular or squamous plaque formation, but they can also be associated with comorbidities such as arthritis, diabetes type II, obesity, and metabolic syndrome [3, 4]. This wide range of psoriasis types indicates that psoriasis is a multifactorial disease where the hereditary factor is determinant. In fact, the first psoriasis related locus (PSORS1) was located in the chromosome 6p21.23 where genes related to HLA-Cw6 are found. In a meta-analysis done with 10,588 psoriatic patients and 22,806 healthy subjects analyzed from three genome-wide association studies (GWAS), thirty-six susceptibility loci were found. The new identified loci included genes whose products are associated with the innate-immune response as interferon-mediated antiviral responses (DDX58), macrophage activation (ZC3H12C), nuclear factor NF*κ*B signaling (CARD14 and CARM1) and genes whose products are involved in the regulation of T-cell function (RUNX3, TAGAP, STAT3, STAT5A, and STAT5B) [5].

**Figure 1:**
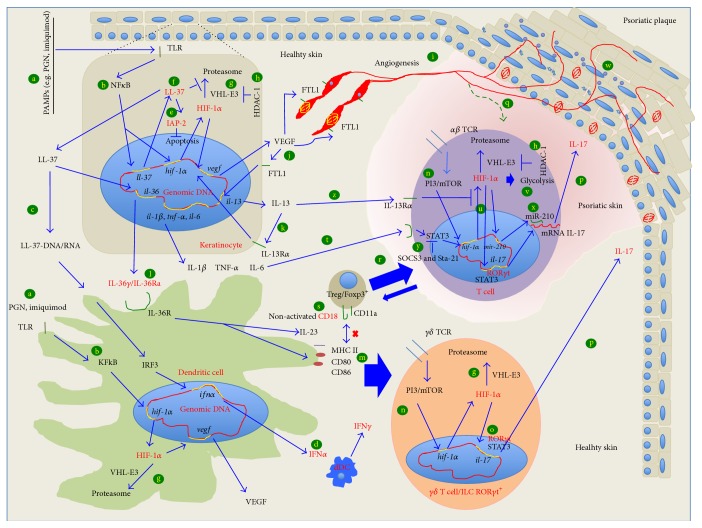
Hypotetical interaction between the keratinocyte and cells from immunologycal system and role of HIF-1*α*, in the generation of a psoriatic lesions. The following mechanism of epidermal response proposed occurs in healthy individuals. But, in psoriatic patients, this mechanism could generate psoriatic lession, because they might have impaired genes involved in the immune homeostasis (pink zone corresponds to psoriatic skin). When virus, bacteria or a fisical factor interacts with epithelial cells, (a) NF*κ*B is activated via TLR (b) and translocated to nucleus to induce LL-37 and HIF-1*α* expression. The released LL-37, together with DNA or RNA (c), activates plasmacytoid dendritic cells to produce IFN*α* and induce the activation of Th17 via IFN*γ* (d). On the other hand, intracellular LL-37 favors proliferation of keratinocytes inducing IAP-2 expression (e); besides, intracellular LL-37 also facilitates angiogenesis inhibiting the proteosomal degradation of HIF-1*α* (f) driven by E3/VHL (g) which is downregulated by HDAC-1 (h). After HIF-1*α* is stabilized by LL-37, HIF-1*α* is translocated to the nucleous to induce VEGF expression, that is, a potent angiogenic factor (i). The concentrations of VEGF also augment in a feedback manner with IL-13/IL-13R system in keratinocytes (j and k). The released LL-37 is also considered as an alarmin that is able to induce IL-36 production (l) which in turn activates dendritic cells and induce IL-23 production (m). The dendritic cells activate *γδ*-T cells and ILC (both ROR*γ*t^+^) located in the healthy dermal epithelium to trigger the production of IL-17 through PI3/mTOR-HIF-1*α*-ROR*γ*t (n, o, and p). The reclusion of Th17 cells is facilitated by the angiogenesis and chemiotatactic molecules in the psoriatic lession (q) and also Th17 could derive from Treg with nonfuntional CD18 (r and s) to produce even more IL-17 (p) via IL-6-Stat3-HIF-1*α*-ROR*γ*t (t, u, and p) in an activated glycolisys (v). IL-17 induces keratinocytes proliferation (w). Some targets proposed for the treatment for psoriasis are shown. The use of miR-210 (x), the use of STAT3 inhibitors: SOCS3 and Sta-21, (y) the downexpression of VHL (g), and the high expression of IL-13R*α* (z).

The skin is an organ that accumulates a high number of cells of the immunological system; for example, a considerable number of T cells reside in normal human skin (approximately twice the number of circulating T cells) and in the epidermis Langerhans cells and CD8^+^ T cells are the most predominant immunological cell types. In the dermis of both mouse and human, dermal dendritic cells (dDCs), macrophages, mast cells, conventional *αβ*T cells, and a small population of type-3 innate lymphoid cells (ILCs) producing IL-17 have been reported. In mice, a particular population of cells called dendritic epidermal T cells (DETCs) are located in the epidermis, and in the dermis the *γδ*T cells (ROR*γ*t^+^) are found (Figure 1, n) [6].

So far, the trigger for psoriasis remains unknown, but it has been suggested that microbial agents or DNA/RNA-LL37 complex delivered by physical factors (UV or dermal damage) activates plasmacytoid dendritic cells (pDC) for the production of IFN*α* that in turn activates dermal dendritic cells (dDCs), migrates to lymph nodes, and produces IFN-*γ*. In the presence of IFN-*γ*, the immune response can be polarized to Th1 and Th17 and activate *γδ*T cells to produce IL-17 who has an important role in the pathology of psoriasis, as this inflammatory cytokine is considered a potent inductor of the keratinocytes proliferation (Figure 1, c and d) [7].

In this review, we will discuss the interaction of HIF-1*α* with cytokines and cells of the immunological system involved in the pathogenesis of psoriasis.

## 2. Psoriatic Animal Models 

Several animal models have been developed to scrutinize psoriasis. In a good psoriasis animal model, the following should happen: keratinocytes hyperproliferation, cellular infiltration, altered vascularity, thickening of epidermis, altered cell differentiation of epidermis, and responsiveness to current antipsoriasis therapies. Within these animal models, there are those involved with genes related to hyperproliferation of keratinocytes: for instance, the transgenic mouse for the “human keratinocytes autocrine growth factor,” amphiregulin [8, 9], and the transgenic mouse for the “peroxisome proliferator-activated receptor,” PPAR *β*/*δ*, both resemble psoriasis because there is an interference in the proliferation and differentiation of keratinocytes [10]. PPAR *β*/*δ* receptor is induced by TNF*α* [11, 12], blocks apoptosis in keratinocytes, contributes to STAT3 phosphorylation, induces angiogenesis, and is upregulated in human psoriatic skin [13, 14]. Besides PPAR *β*/*δ* directly induces the differentiation of keratinocytes, and in the transgenic mouse model a light augment of Th17 is also observed [15].

Several murine psoriasis models have been generated with a dysfunction in a transcription factor that regulates the expression of innate immunity molecules. For instance, deletion of IKK*β*, which mediates canonical NF*κ*B activation, and transgenic expression of mutated I*κ*B*α* produce a fulminant psoriasis-like disease in mice with TNF-dependent action [16, 17].

Recently, Grinberg-Bleyer et al. described a new murine psoriasis model that lacks the expression of p65 or c-Rel in cells of the epidermis. Those mice lacking both subunits developed severe dermatitis after birth which is resolved 30 days after birth by the effect of Treg cells, but when Treg cells were eliminated using anti-CD25 antibodies the deficient mice exhibited worsened pathology, and the symptoms were reversed with anti-TNF*α* treatment (Figure 1, b) [18].

The dysfunction in the activity of other transcription factors, for instance, AP-1 and STAT3, also contributes to the initiation of skin inflammation. Mice with deficient expression of JunB and its functional coworker c-Jun, as well as mice with overexpression of FOS (all components of AP-1), generate a phenotype resembling the histological and molecular hallmarks of psoriasis, including arthritic lesions. Furthermore, epidermal keratinocytes of psoriatic patients have the JunB expression reduced, in comparison with cells from healthy subjects [19]. Besides, STAT3 transgenic mice and SOCS3 knockout mice (the negative regulator of STAT3) have constitutive activation of STAT3 and both develop murine IL-6-driven psoriasis [20, 21]. (Figure 1, t and y).

Other sorts of psoriatic animal models include those where cytokines and cells of immune system are involved. The role of type I interferons in the psoriasis was demonstrated in mice deficient to “IFN regulatory factor-2” (IFNR-2), a transcriptional repressor for IFN-*αβ* signaling (Figure 1, c). These mice developed a skin disease similar to human psoriasis [22]; in fact, now, we know that type I interferons promotes the activation of dDCs [23].

Another cytokine involved in the pathological mechanism of psoriasis is IL-36, a submember of IL-1 family. The overexpression of IL-36*α* in transgenic murine keratinocytes promotes acanthosis, hyperkeratosis, cells infiltration, and increased expression of cytokines and chemokines. The deficiency of IL-36Ra, the natural antagonist of this cytokine, increases the severity of the lesions in the epidermis. Additionally, mice deficient in IL-36 or in its receptor IL36R are protected from psoriasiform dermatitis induced by imiquimod [24]. Also it is known that IL-1*β*, TNF*α*, and IL-36 activate dDCs and induce the production of IL-23 that is necessary for naive T cells to polarize to Th17, suggesting that IL-23 could be the link between the innate and adaptive immune response that occurred in the psoriatic lesions (Figure 1, l and m) [25].

In 2009, a transient model of psoriasis-like disease was reported, induced in healthy mice (nongenetically modified) with the use of a TLR7 and TLR8 ligand (imiquimod). This model showed the described skin lesions of psoriasis pathogenesis, including activation of pDC and the dependence on Th17 cells producing IL-17A, IL-17F, and IL-22 (Figure 1, c and d) [26].

The deficient regulation in the cellular response is also involved in the development of psoriasis. In normal conditions, T regulatory (Treg) cells regulate the activity of autoreactive Th1 and Th17 cells but in psoriasis it has been suggested that Treg cells might not be functional, and this was evident in the CD18^hypo^ mouse model. Homozygous mice PL/J CD18 hypomorphic (CD18^hypo^) developed spontaneously a psoriasis-like skin disease after 12–14 weeks of age [27]. CD18 is a molecule that together with CD11a forms an adhesion molecule of the *β*2 integrin family, important for the complete function of Treg cells. It has been suggested that CD18^hypo^ mice induce psoriasis because Treg cells with low expression of CD18, or with a not fully active molecule, cannot regulate the activity of autoreactive Th1 and Th17 cells (Figure 1, s) [28].

The altered function of angiogenic molecules also produces psoriasis. “Vascular endothelial growth factor” (VEGF-) transgenic mice [29], “endothelial specific receptor tyrosine kinase” (K5-Tie2-) transgenic mice [30], and “transforming growth factor beta 1” (K5-TGFb1-) transgenic mice [31] are psoriasis animal models that highlight the importance of angiogenesis in this pathology. Tie2 is the receptor of angiopoietin that together with VEGF is essential for proliferation, maturation, and maintenance of blood vessels (Figure 1, i). In both models, the hyperproliferation of keratinocytes and the abundance of immunological cells infiltrate, including Th17 cells, are detected in the psoriasiform lesions. The overexpression of VEGF not only can be promoted by TGF*β* but also can be regulated by HIF-1*α* (Figure 1, j), as it is overexpressed in the psoriatic skin.

## 3. HIF-1***α*** Regulation and Function

“Hypoxia inducible factor” (HIF) transcription factor family is integrated by 3 alpha molecules and one beta molecule, called HIF-1*α*, HIF-2*α*, HIF-3*α*, and HIF-1*β*. HIF-1*α* has been the most studied one because in altered biological processes where the cells are in hypoxic conditions, such as cancer, HIF-1*α* is overexpressed and translocated to nucleus where it interacts with HIF-1*β* and p300 cofactors to induce transcription of hypoxia-related target genes [32].

In cells of normal tissues and in normoxic conditions, HIF-1*α* is constitutively expressed in the cytosol and its activity is regulated by degradation in the proteasomal system (Figure 1, g) [33]. In myeloid cells and T cells, the expression of HIF-1*α* can also be induced via TLR-IKK-NF*κ*B and PI3K-mTOR-TCR, or dectin1-Akt-mTOR (Figure 1, n) [34, 35]. In the cytosol, HIF-1*α* is hydroxylated by “prolyl hydrolases” (PHD2) in its Pro402 and Pro564 residues for its regulation [36, 37]. The hydroxylated residues are recognized by the “von Hippel-Lindau” (VHL) E3 ubiquitin ligase complex (containing elongin B and elongin C, Cul2, and Rbx1), and HIF-1*α* is ubiquitinated and hydrolyzed by the proteasome (Figure 1, g) [38, 39].

Protein SSAT2 stabilizes the union between VHL-E3 ubiquitin ligase complex and HIF-1*α* promoting HIF-1*α* degradation [40]. HIF-1*α* can be acetylated in the Lys532 by ARD1 for its subsequent degradation via VHL [41], but the “metastasis-associated protein 1” (MTA1) can counteract the activity of ARD1 [42]. “VHL-interacting deubiquitinating enzyme 2” (VDU2) is another protein that stabilizes HIF-1*α*, because it deubiquitinates HIF-1*α* avoiding its degradation [43, 44]. It is important to note that HIF-1*α* degradation is not only carried out by VHL-E3 ligase; the “receptor of activated protein kinase C” (RACK1) [45, 46], SSAT1, HSP70, and COMMD1, as well as the “hypoxia-associated factor” (HAF), interact with HIF-1*α* and elongin C mediating the degradation of HIF-1*α*. However, HSP90 [46], SEPT9_v1 [47], and Jun activation domain-binding protein-1 (Jab1) compete for the interaction with HIF-1*α* to prevent its proteasomal degradation [48].

On the other hand, in hypoxia conditions, the degradation of HIF-1*α* is interrupted because the PHDs are inactivated by the mitochondrial reactive oxygen species (ROS) leading to the accumulation HIF-1*α* in the cytosol [49, 50]. Under hypoxia, HDAC-1 and HDAC-7 are induced; meanwhile HDAC-1 downregulates the expression of VHL favouring the stabilization of HIF-1*α* (Figure 1, h) [51, 52] and HDAC-7 binds to HIF-1*α* to cotranslocate to the nucleus [53].

In the nucleus, HIF-1*α* binds to HIF-*β* for its transcriptional activity, being improved when they are associated with the coactivators p300/CBP, SRC-1, and TIF2 [54], regulator associated protein of mTOR, “orphan nuclear receptor estrogen-related receptor” (ERRs), and Thiol-redox regulator [55]. Nevertheless, if HIF-1*α* is translocated when is hydroxylated at the asparagine 803 residue by the asparaginyl hydroxylase FIH-1, it cannot bind with the cofactors, and the transactivation of HIF-1*α* is abolished [56].

The function of HIF-1*α* can also be regulated in the nucleus; the proteins Necdin [57], “testis specific gene antigen 10” (TSGA10) [58], COMMD1 [59], p14^ARF^ tumor suppressor protein [60], and SIRT1 [61] can interact with HIF-1*α* inhibiting its transcriptional activity.

The list of target genes of HIF-1*α* is very large; several evidences have implicated HIF-1*α* in the metastasis of tumoral cells. The reports show that the expression of intercellular adhesion molecules (*α*_5_ *β*_3_, *α*_5_ *β*_5_, *β*1 integrins, and E-cadherin) [62–65] “matrix metalloproteinases” (MMP2 and MP9) [66, 67] and chemokines (CXCR4, c-Met, and CCR7) is regulated by HIF-1*α* [68–70]. In angiogenesis, HIF-1*α* has been implicated in the regulation of VEGF, calcitonin receptor-like receptor, Sema4D, “stem cell factor” (SCF), and angiopoietin between others that will be discussed below [71, 72]. In apoptosis, HIF-1*α* also plays important roles; it regulates the expression of BNIP3 (which is a member of Bcl-2 family), interacts with p53, and regulates the expression of Puma, Bax, and p21 [73]. Besides, HIF-1*α* promotes the cellular undifferentiation of various stem cell populations; for example, not only HIF-1*α* interacts with Notch blocking the differentiation of neuronal and myogenic progenitors, but also HIF-1*α* induces the expression of erythropoietin (EPO), which is necessary for the differentiation of red blood cells [74]. In the energy metabolism, HIF-1*α* regulates the expression of glucose transporters GLUT1 [75] and GLUT3 [76], 6-phosphofructo-2-kinase, phosphoglycerate kinase 1 (PGK1), and pyruvate kinase M2 that regulate the glycolytic flux [77].

Recently, it was reported that HIF-1*α* is also involved in the differentiation of Treg and Th17 cells [78, 79].

## 4. HIF-1***α*** and the Angiogenesis of Psoriasis

As previously described, HIF-1*α* is a transcription factor that acts over several target genes, but HIF-1*α* plays an important role in the expression of proangiogenic genes implicated in psoriasis, such as “vascular endothelial growth factor” (VEGF) [80–83], VEGF receptors FLT-1 [83] and FLK-1 [84], “plasminogen activator inhibitor-1” (PAI-1) [85], angiopoietins [86], TIE-2 [86], “matrix metalloproteinases” MMP-2 and -9 [87] calcitonin receptor-like receptor, Sema4D [71, 72], and “cytokine stem cell factor” (SCF) [88].

In normal skin HIF-1*α* is expressed in the epidermis cells, as the skin has been suggested to be a hypoxic tissue [89], but in psoriatic lesions such expression is increased [90], and as a consequence proangiogenic mediators, such as VEGF, IL-8 and angiopoietins, are augmented in the psoriatic skin [91, 92].

The importance of VEGF in psoriasis is clearly seen in VEGF transgenic mice that develop skin lesions that resemble psoriasis [29]. As the presence of* Staphylococcus aureus* in the skin of psoriatic individuals has been described [93], our group studied the effect of peptidoglycan (PGN) from these bacteria in keratinocytes. We found that keratinocytes stimulated with PGN from* S. aureus* induce the overexpression of LL37 and VEGF (Figure 1, f, c and j), but downexpressed VHL (Figure 1, g) [94]; furthermore, when we transfected keratinocytes with* ll-37* VEGF and IAP-2 they were highly expressed (Figure 1, j and e) suggesting that this antiapoptotic molecule could favor keratinocytes proliferation in psoriasis and could participate in the VEGF expression to promote angiogenesis [95]. Li et al. reported that the angiogenic activity of porcine PR39 (homologous to human LL-37) occurs because PR39 inhibits the proteasomal degradation of HIF-1*α*, and, in turn, it increases the expression of VEGF (Figure 1, f).

Similar to Chamorro et al. [96], who shows that LL-37 had antiapoptotic effect over keratinocytes, we reported recently that PGN has an antiapoptotic effect over HaCaT cells treated with TNF*α* through the production of LL-37 and IAP-2 (Figure 1, e) [97].

Studying the regulation mechanisms for the VEGF production in keratinocytes, we found that HaCaT cells transfected with* hdac-1* highly expressed VEGF via HIF-1*α* (Figure 1, h), and HaCaT cells transfected with* vhl* scarcely produced VEGF (Figure 1, g), but the low production of VEGF could be counteracted by* hdac-1* cotransfection. (Figure 1, g and h) [52]. These assays showed the direct and opposed effect of HDAC-1 and VHL over VEGF production via HIF-1*α* (Figure 1, g and h). When we analyzed the expression of those molecules in psoriatic skin, we found that HDAC-1, HIF-1*α*, and VEGF are overexpressed, but not VHL, suggesting that the absence of VHL favors HIF-1*α* activity and thus angiogenesis in psoriatic skin [90].

On the other hand, the dermis of psoriatic skin is infiltrated predominantly by IFN*γ*-producing Th1 cells and IL-17-producing Th17 cells [98]. It has been reported that IL-17A has proangiogenic effects but in a HIF-1*α*-independent pathway; however, at the same time, IL-17A can stimulate the expression of proangiogenic factors in fibroblast and keratinocytes, including VEGF, feeding back the angiogenesis in psoriasis (Figure 1, p) [99–101].

Angiopoietins Ang-1 and Ang-2, and their receptor Tie-2, are involved in the angiogenic process of psoriasis and all are induced by HIF-1*α* [102]. Ang/Tie-2 is essential for the growth, maturation and stabilization of blood vessels and, in the papillary dermis of psoriatic skin, Ang-1, Ang-2, and Tie-2 are overexpressed [103].

The prominent reduction of Ang-2 expression after successful therapy suggests an important role of Ang-2 in psoriasis [102]. In a transgenic mouse model, the overexpression of Tie-2 causes a psoriasis-like disease, and interestingly the repression of Tie-2 in transgenic mice reversed the disease completely [104].

## 5. Role of HIF-1***α*** in the Th17 Polarization and in the Psoriasis

Since 1986, when Mosmann et al. demonstrated the existence of two different kinds of helper T cells clones (Th), named T helper cell 1 (Th1) and T helper cell 2 (Th2) [105], different groups have described the effect of these subsets in several pathologies. Initially, the psoriasis was considered as a disease with Th1 phenotype [106, 107]; however, this did not fully explain how Th1 cells mediated the tissue damage in the chronic inflammation [108]. A new hypothesis arose to explain the psoriasis' phenotype with the description of a new subpopulation of T helper cells called Th17 cells, IL-17 producers [109]. The main physiological role of Th17 cells has been shown in mucosal and epithelial host defence, especially against fungal infections and extracellular bacteria [110], but Th17 cells have also been related to the development of autoimmune and inflammatory diseases [111]. IL-17 induces the production of IL-6, IL-8, “granulocyte-colony-stimulating factor” (G-CSF), prostaglandin E2 (PGE), and “leukemia inhibitory factor” (LIF) in epithelial, endothelial, and fibroblastic cells [112]. In particular, in the pathophysiology of psoriasis, Th17 cells induce the production of IL-6 and IL-8 in keratinocytes [113]. Systemically, serum levels of IL-17 and of other proinflammatory cytokines correlate with psoriasis severity [114]. Similarly, Th17, Th1, and Th22 cells (another subpopulation of Th cells characterized by IL-22 production) are increased in psoriatic lesions and in blood circulation [115, 116]. According to this, IL-17 acts as a proinflammatory cytokine that amplifies the development of cutaneous inflammation, keeping keratinocytes in constant stimulation for the production of more proinflammatory cytokines, in a feedback loop mode that keeps the chronic inflammation as an important characteristic of psoriasis.

CD4^+^CD25^high^Foxp3^+^ Tregs or natural Treg cells are typically considered inhibitors of autoimmune responses, suppressing Th1 and Th17 activities. Similar to Th17 subpopulation, Treg cells are increased in the peripheral circulation and in skin tissue lesions of psoriatic patients [117]. To explain why inflammation is not resolved in the psoriatic skin even if Treg are also increased, two situations has been suggested; the function of Treg cells is impaired in psoriasis, or Treg cells can differentiate into inflammation-associated Th17 cells under inflammatory conditions. In the CD18^hypo^ PL/J murine psoriasis model, the reduced expression of CD18/*β*2 integrin is associated with progressive Treg dysfunction. Recently,* in vivo* studies has reported that Treg cells derived from CD18^hypo^ PL/J mice have high propensity to differentiate to Th17 cells, and* in vitro* studies showed that the addition of CD18 neutralizing antibodies to Treg-dendritic cells cocultures promoted the switch of CD18(wt) PL/J Treg cells to Th17 cells in a dose-dependent manner, similar to the conversion rates observed* in vivo* in the CD18^hypo^ PL/J Treg cells (Figure 1, r) [28].

Shi et al. were the first to report that, for the Th17 differentiation, the induction of glycolytic enzymes (Figure 1, v) is required for the production of HIF-1*α* via mTOR (Figure 1, n); they observed that when glycolysis and mTOR were blocked, the Th17 differentiation was inhibited, and Treg cells generation was promoted [78]. Today, it is known that, for Th17 differentiation, cytokines IL-6 and TGF-*β* are necessary to induce naïve T cells to proliferate to Th17 phenotype, while cytokines IL-21 and IL-23 are important for the maintenance and survival of the lineage [118–120].

The characteristic transcription factors that act for the production of IL17 cells are “retinoic acid receptor-related orphan receptor” alpha and gamma (ROR*α* and ROR*γ*t) [121, 122] and STAT3 [123, 124]; they interact with HIF-1*α* for IL-17 expression. Dang et al. showed that in a hypoxic and inflammatory environment (IL-6), HIF-1*α* leads the Th17 cell differentiation together with STAT3 (Figure 1, t). This was suggested because, in the presence of IL-6 and TGF-*β*, Stat3^−/−^ T cells did not produced HIF-1*α*, nor IL-17, and when naïve T cells were transfected with* hif-1α*, they produced more IL-17 compared with nontransfected cells; furthermore they showed that HIF-1*α* ^−/−^ T cell did not differentiate to Th17 cells in hypoxic conditions, but instead they differentiated to Treg cells. Dang et al. suggested that in naïve T cells STAT3 induces the production of HIF-1*α* in the presence of IL-6, and HIF-1*α*-STAT3 induces the production of ROR*γ*t. ROR*γ*t recruited with STAT3, HIF-1*α*, and p300 binds to the IL-17 promoter for IL-17 transcription. In the same work, Dang et al. showed the importance of HIF-1*α*/STAT3 in autoimmunities; HIF-1*α* ^−/−^ mice deficient for IL-17 were more resistant to experimental autoimmune encephalomyelitis induced with “myelin oligodendrocyte glycoprotein peptide” (MOG35–55) [79]. In this point, it is important to remember that transgenic mice to STAT3 and the knockout mice to the “suppressor of STAT3” (SOCS3) developed squamous skin that resembles human psoriasis, and possibly the high expression of STAT3 favored the Th17 differentiation but at the time the assays were not performed [20, 21]. Now, it is known that STAT3 and STAT5 bind to the same DNA sites in the IL-17 promoter, but they have opposing regulations [125]; meanwhile STAT3 promotes Th17 cells and STAT5 promotes Treg cells. In the absence of IL-6, IL-2 induces the expression of Stat5 in Th17 cells that is a key transcription factor for the development of CD4^+^ FOXP3 regulatory T cells [126]. Subjects with deficiency in the production of IL-2 are susceptible to autoimmune diseases [79, 125]. The presence of cytokine IL-6 or IL-2 defines which STAT is going to be acting, as IL-2 is a high inductor of STAT5 and IL-6 promotes the production of STAT3.

The activity of Th17 is also regulated by IL-13 (Figure 1, z). In contrast with Th0, Th1, or Th2 cells, polarized Th17 cells have high expression of IL-13R (integrated by the dimer IL-13R*α*/IL-4R) [127]. IL-13 and IL-4 attenuate IL-17 production in Th17 cells from wild type but not from IL-4R knockout mice [128]. Our group reported high expression of IL-13R, of both IL-13R*α* and IL-4R mRNA, in human psoriatic skin [129]. Furthermore, we also showed that keratinocytes are IL-13 producers when stimulated with rVEGF or with PGN via LL-37-HIF-1*α*-VEGF (Figure 1, j and k) [94]. These results suggest that in the psoriatic skin HIF-1*α* favors the polarization of Th17 cells and induces VEGF for angiogenesis, but at the same time VEGF could also lead the control of the inflammatory environment by the induction of IL-13 (Figure 1, j) to counteract the activity of Th17 cells (Figure 1, z). Nevertheless, we also reported that IL-13R protein was not increased as observed for its mRNA in the psoriatic skin, suggesting that the protein is not available for the inhibition of Th17 cells and it could be the cause why in psoriatic patients the lesions are not controlled [129].

On the other side, the plasticity of Treg cells to become Th17 cells has been studied in human psoriasis and in mouse models. Positive cells to both Th17 and Treg populations (IL-17A^+^/Foxp3^+^/CD4^+^) have been found in CD18low P/J mouse and in skin lesions from patients with severe psoriasis. Treg cells from psoriatic patients had enhanced propensity to differentiate to IL-17A-producing cells in cells stimulated* ex vivo* with IL-23 (cytokine strongly associated with the psoriasis, Figure 1, m), but, interestingly, the treatment with trichostatin-A, a “histone/protein deacetylase” (HDAC) inhibitor prevented the cellular differentiation [130]. These data suggest that HDAC favors the production of IL-17 in patients with psoriasis (Figure 1, h). Our group has demonstrated that in keratinocytes transfected with* hdac-1* HIF-1*α* translocated to the nuclei and the production of VEGF was induced, but the treatment with trichostatin-A inhibited that process. It is known that HDAC-1 induces the downexpression of VHL which leads HIF-1*α* to proteasomal degradation [51, 52]. Perhaps in the psoriasis Treg cells treated with trichostatin-A occurred that when HDAC-1 was inhibited, the expression of VHL augmented, HIF-1*α* was proteolyzed (Figure 1, h), and the expression of IL-17 decreased, giving as result that Treg cells could not switch to Th17. In the skin of psoriatic skin, we found that when VHL was not detected, HIF-1*α* and VEGF were highly expressed [90].

## 6. HIF-1***α*** as a Potent Therapy Target in Psoriasis

All the results shown above suggest that HIF-1*α* could be a target to control psoriasis considering that this transcription factor is involved in angiogenesis and in Th17 differentiation.

Actually, there are few works that focused on the blockade of HIF-1*α* as treatment for inflammatory diseases. Kim et al. reported that the expression of “extra cellular superoxide dismutase” (EC-SOD) is downregulated in the skin of patients with psoriasis in comparison with healthy subjects. In HaCaT cells overexpressing EC-SOD there was inhibition of HIF-1*α* production under hypoxia conditions and in EC-SOD transgenic mice irradiated with UV a decreased inflammation and angiogenesis was seen [131].

The polyphenol “epigallocatechin-3-gallate” (EGCG), obtained from green tea, is another molecule that acts over HIF-1*α*. In arthritis model (IL-1RaKO) EGCG decreased the arthritis index and showed protective effects against joint destruction. The expression of proinflammatory cytokines, oxidative stress proteins, p-STAT3 (Y705), and p-STAT3 (S727), mTOR, and HIF-1*α* were significantly lower in mice treated with EGCG. Besides, the proportion of Foxp3^+^ Treg cells was increased in the spleens of mice treated with EGCG, whereas the proportion of Th17 cells was reduced.* In vitro*, p-STAT3 (Y705) and p-STAT3 (S727) and HIF-1*α* and glycolytic pathway molecules were decreased by EGCG [132].

Another strategy for the control of HIF-1*α* is the use of microRNAs. The microRNA miR-210 downregulates HIF-1*α* production, and the deletion of miR-210 promotes Th17 differentiation under limited oxygen conditions. In experimental colitis, miR-210 reduced the abundance of HIF-1*α* transcripts as well as the number of cells that produce inflammatory cytokines, controlling the disease severity (Figure 1, x) [133].

Decoy oligonucleotides have also been used as a strategy for treatment. In K5.Stat3C mice, where keratinocytes express a constitutively active Stat3 and develop psoriasis-like skin lesions, Stat3 was targeted with a decoy oligonucleotide and after treatment psoriatic lesions were reversed [21].

STA-21, a small Stat3 inhibitor, (Figure 1, y) was also evaluated in K5.Stat3C mice. Treatment with STA-21 markedly inhibited the cytokine-dependent nuclear translocation of Stat3 in normal human keratinocytes* in vitro*. Topical application of STA-21 abolished the generation of skin lesions in K5.Stat3C mice. STA-21 was also evaluated in patients. Psoriatic lesions in six of the eight patients showed improvement after topical STA-21 treatment for 2 weeks [134].

We suggest that these molecules could be used to control the psoriasis pathology as they act over HIF-1*α* to control IL-17 production and angiogenesis development.

## 7. Conclusion

HIF-1*α* is a transcription factor highly regulated that has an important role in the angiogenesis and in the generation of Th17 cells in the skin of psoriatic patients. HIF-1*α* and its regulators (HDAC-1, HDAC-7, VHL-E3, PHD2, LL-37, HIF-1*β*, mTOR, miR-210, ROR*γ*t, STAT3, and IL-13Ralpha, as well as glycolysis enzymes) could be important pharmacological targets to restore the lack of regulation in the angiogenesis and in immunological processes involved in psoriasis.
